# Energy budget and carbon footprint in a wheat and maize system under ridge furrow strategy in dry semi humid areas

**DOI:** 10.1038/s41598-021-88717-3

**Published:** 2021-04-30

**Authors:** Changjiang Li, Shuo Li

**Affiliations:** 1 Hainan Key Laboratory for Sustainable Utilization of Tropical Bioresources, College of Tropical Crops, Hainan University, Haikou, 570228 China; 2 School of Life Sciences, Institute of Life Science and Green Development, Hebei University, Baoding, 071002 China; 3 College of Agronomy, Northwest A&F University, Yangling, 712100 China

**Keywords:** Environmental sciences, Agroecology, Agroecology

## Abstract

The well-irrigated planting strategy (WI) consumes a large amount of energy and exacerbates greenhouse gas emissions, endangering the sustainable agricultural production. This 2-year work aims to estimate the economic benefit, energy budget and carbon footprint of a wheat–maize double cropping system under conventional rain-fed flat planting (irrigation once a year, control), ridge–furrows with plastic film mulching on the ridge (irrigation once a year, RP), and the WI in dry semi-humid areas of China. Significantly higher wheat and maize yields and net returns were achieved under RP than those under the control, while a visible reduction was found for wheat yields when compared with the WI. The ratio of benefit: cost under RP was also higher by 10.5% than that under the control in the first rotation cycle, but did not differ with those under WI. The net energy output and carbon output followed the same trends with net returns, but the RP had the largest energy use efficiency, energy productivity carbon efficiency and carbon sustainability among treatments. Therefore, the RP was an effective substitution for well–irrigated planting strategy for achieving sustained agricultural development in dry semi-humid areas.

## Introduction

The well-beings of both human and other organisms on earth are in danger due to the ongoing environmental degeneration^1^. The increasing greenhouse gas (GHG) emission from artificial disturbance is deteriorating the environmental quality^2^. Annual GHG emissions in both agricultural and natural ecosystems are up to ~ 5.9 Gt carbon dioxide equivalent (CO_2_–eq) per year (1 Gt = 10^9^ t)^3^. In China, the GHG emissions from agricultural soils are approximately 686 Mt CO_2_–eq (1 Mt = 10^6^ t), accounting for 9.2% of the nation's total in 2007^4^. Moreover, the manufacture, transport and application of fertilizers and pesticides, power use for irrigation, and field operations all require fossil fuels, the combustion of which results in large GHGs emissions^5,6^. Hence, it is vital to reduce GHG emissions from farming and related activities to alleviate climate change, and to resolve related environmental issues.

As a quantitative indicator of GHG emissions, the carbon footprint (CF) has gained widespread popularity and application in agricultural production due to its special functions of identifying eco–friendly production systems^7^. The relationship of both energy input and output, energy use efficiency, energy productivity, and specific energy are also valuable indicators for screening a cleaner production system and mitigating GHG emissions^1,8^. Recently, increasing research has focused on the CF and energy performance in diverse agricultural systems, such as the mono–cropped production of wheat^4^, maize^9^, and rice^1^, as well as the winter wheat (*Triticum aestivum* L.)–summer maize (*Zea mays* L.) double cropping system^10,11^. Those studies are mainly based on tillage, which is an energy–intensive field operation that contributes to 30% of total energy use in agricultural production^12^. Consequently, a shift in field management practices is urgently required with high energy use efficiency and low GHG emissions for grain production with environmental sustainability^1^.

The energy consumption derived from irrigation is one of the most important GHG sources^13^. Adopting water–saving management strategies is also an efficient measure for achieving sustained agricultural production in arid, semi–arid, and even dry semi-humid areas^14,15^. As an innovative water–saving technology, the ridge–furrow with plastic film mulching on the ridge (RP) has the advantages of building ridges along the farmland contours to reduce soil and water loss from heavy rains, penetrating collected light-rain water into deep soil and preserving soil moisture in decreasing unproductive evaporation, and thus prolongs the period of soil water availability to plants^16^. Several field studies also have identified that the RP could increase the water use efficiency and crop yields in dry semi–humid areas^17,18^. It is noteworthy that RP could increase the indirect GHG emissions because of plastic film production, marketing and use in the field, meanwhile, cause farmland environment pollution^19,20^. However, whether RP is suitable for semi–humid areas to decrease energy consumption, GHG emissions, and economic benefits of production remains unknown.

The current experimental site is in the southern area of the Loess Plateau, one of the major dry semi-humid farming areas of China, which spreads over approximately 64 million hectares and supports nearly 100 million people^21^. The typical intensive winter wheat–summer maize system produces approximately 60% of the total cereal production of Shaanxi Province^22^. However, the high grain yields are achieved at the expense of excessive groundwater consumption, which has been hindering the sustained agricultural production^23^. Additionally, this issue is becoming increasingly severe with the acceleration of industrialization and urbanization^24^. Although the RP has been recommended in dry semi-humid areas, it was mainly performed in the mono–cropped production of wheat^25^, maize^16,26^, and foxtail millet^18^. It is unknown that whether RP is suitable for the intensive winter wheat-summer maize system with high energy use efficiency and economic benefits to promote the sustained agricultural production in this region. To fill this knowledge gap, the main objectives of this study are to (i) evaluate the economic feasibility of the RP; (ii) compare the energy use and CF of the RP with conventional rain-fed flat planting and well-irrigation planting strategies; and thus (iii) identify whether is the RP suitable for achieving sustained agricultural production under a highly intensive wheat–maize cropping system or not.

## Results

### Productivity and economics

**Figure 1:**
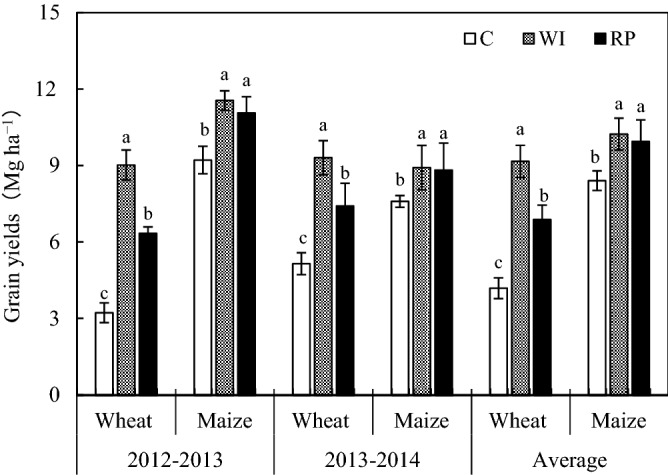
Effect of different planting strategies on grain yields during wheat and maize periods. C, conventional rain–fed flat planting; WI, well-irrigation planting; RP, ridge-furrow planting with plastic film mulch over the ridge^23,52^. The same in subsequent figures and tables. Bars are standard error values. Different lowercase letters over error bars indicate significant difference during the same crop growth period at *P* < 0.05. The same in subsequent figures.

The wheat and maize grain yields ranged from 4.18 to 9.16 Mg ha^−1^ season^−1^ to 8.40–10.23 Mg ha^−1^ season^−1^ during the two rotation cycles (Fig. 1). The WI and RP strategies significantly increased grain yields of both wheat (119.0% and 64.4%, respectively) and maize (21.8% and 18.3%, respectively) relative to those under the control. The average annual wheat yield was significantly lower by 24.9% under RP than that under WI, whereas no significant difference was observed between the WI and RP strategies.

**Figure 2:**
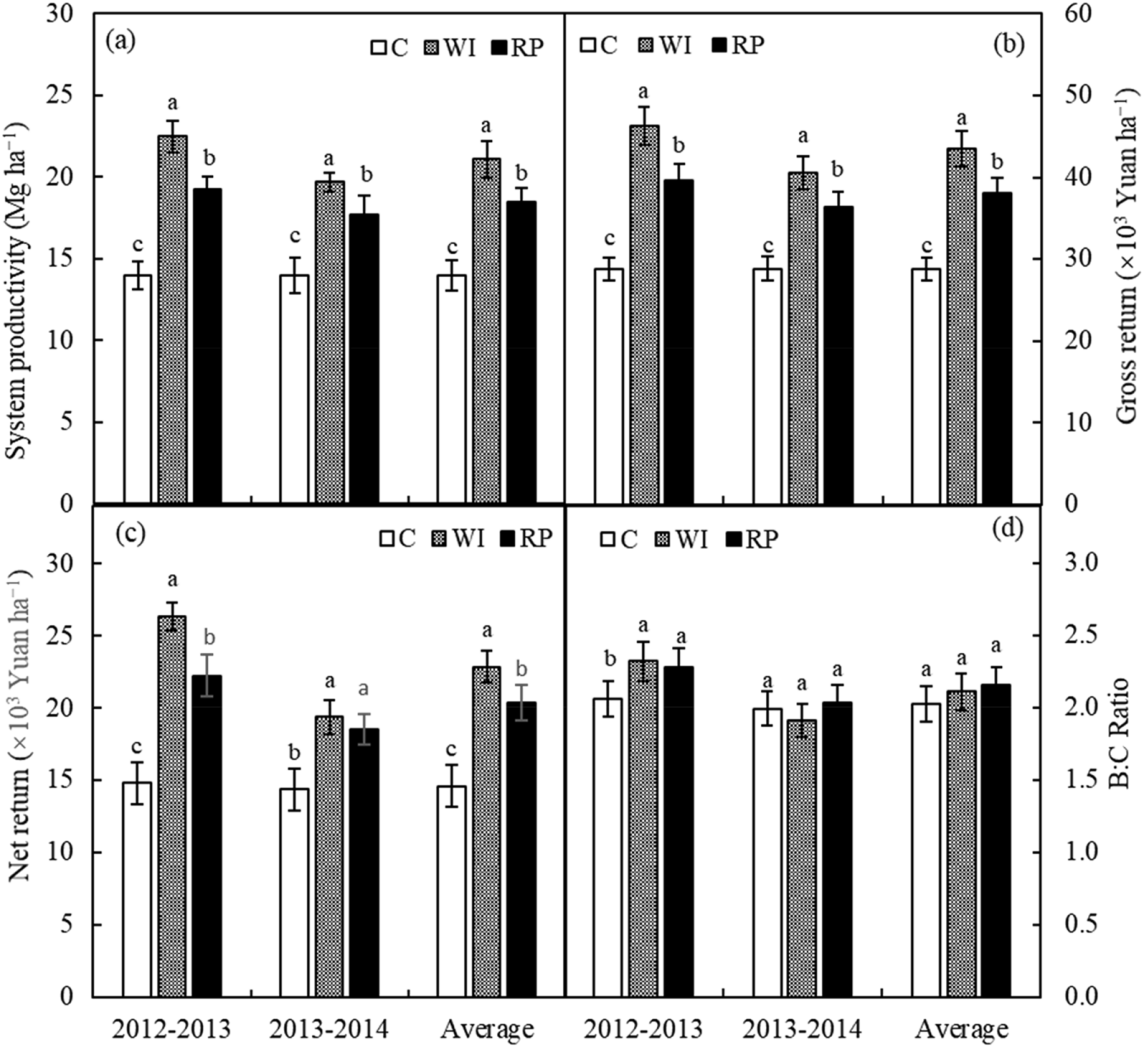
Effect of different planting strategies on system productivity (**a**), gross return (**b**), net return (**c**) and benefit: cost ratio (B:C ratio), (**d**) of wheat–maize cropping system.

Across the 2 rotation cycles, the WI and RP improved the system productivity by 50.9% and 32.1%, respectively, relative to those under the control (Fig. 2a). The average annual gross return and net return ranged from 28.78 to 43.44 × 10^3^ Yuan ha^−1^ to 14.59–22.86 × 10^3^ Yuan ha^−1^ with the trends of C < RP < WI (Fig. 2b,c). The average annual benefit: cost ratio was 2.03, 2.11 and 2.16 under the control, WI and RP strategies, and no significant difference existed between each strategy for the benefit: cost ratio during the two rotation cycles (Fig. 2d).

The total costs of wheat and maize production ranged from 6.266 to 10.466 × 10^3^ Yuan ha^–1^ season^−1^ to 8.276–10.076 × 10^3^ Yuan ha^–1^ season^−1^, and also fell in the same trends of C < RP < WI during the two rotation cycles (Table 1). Regarding the entire rotation cycle of wheat and maize, the total cost was up to 17.017 × 10^3^ Yuan ha^–1^ under RP, which was higher by 17.0% than that under the control, and was lower by 17.2% than that under WI. The costs derived from the use of machinery (6.45 × 10^3^Yuan ha^–1^ yr^–1^) occupied 44.4% and 31.4% of the total costs of crop production under the control and WI, but increased up to 7.65 × 10^3^Yuan ha^–1^ yr^–1^ under RP. The costs derived from irrigation (1.125 × 10^3^Yuan ha^–1^ yr^–1^) accounted for 7.7% of total costs of crop production under the control, but it increased by 3.75 × 10^3^ Yuan ha^–1^ yr^–1^ under WI and reduced by 0.225 × 10^3^ Yuan ha^–1^ yr^–1^ under RP. The inputs of labour consumed 2.175 × 10^3^ Yuan ha^–1^ yr^–1^ under both strategies of the control and RP with an increase of 2.25 × 10^3^ Yuan ha^–1^ yr^–1^ under WI. The costs derived from the use of seeds, fertilizer, and plant protection (including herbicide, insecticide, and fungicide) were 1.14 × 10^3^, 2.752 × 10^3^, 0.9 × 10^3^ Yuan ha^–1^ yr^–1^ in every strategy. A cost of 1.5 × 10^3^ Yuan ha^–1^ yr^–1^ was also expended under RP.

**Table 1:** Effect of different planting strategies on annual average cost (Yuan ha^–1^) of cultivation of wheat–maize cropping system.

Particulars	Wheat period			Maize period			The entire rotation cycle		
	C	WI	RP	C	WI	RP	C	WI	RP
Seeds	390	390	390	750	750	750	1140	1140	1140
Farm machinery	2700	2700	3300	3750	3750	4350	6450	6450	7650
Irrigation	375	3000	300	750	1875	600	1125	4875	900
Fertilizer	1376	1376	1376	1376	1376	1376	2752	2752	2752
Plant protections	450	450	450	450	450	450	900	900	900
Plastic film	0	0	750	0	0	750	0	0	1500
Labor	975	2550	975	1200	1875	1200	2175	4425	2175
Total	6266	10,466	7541	8276	10,076	9476	14,542	20,542	17,017

C, conventional rain–fed flat planting; WI, well-irrigation planting; RP, ridge-furrow planting with plastic film mulch over the ridge.

### Energy budget

The annual energy inputs of wheat production were 28,395, 60,255, and 34,102 MJ ha^−1^ under the control, WI, and RP, respectively (Table 2). The energy inputs from irrigation occupied 59.6% of total energy inputs of wheat production under WI, but it accounted only for 14.7% under both the control and RP. Additionally, the energy inputs of fertilizers and machinery contributed 53.9% and 21.2% under the control, and contributed 44.9% and 21.0% under RP, to the total energy inputs for wheat production. Meanwhile, the use of plastic film contributed 10.7% to the total energy inputs for wheat production.

**Table 2:** Effect of different planting strategies on annual average energy inputs and outputs (MJ ha^–1^) of wheat–maize cropping system.

Particulars	Wheat period			Maize period			The entire rotation cycle		
	C	WI	RP	C	WI	RP	C	WI	RP
**Input**									
Seeds	2355	2355	2355	339	339	339	2694	2694	2694
Farm machinery	6022	6022	7161	4344	4344	7247	10,366	10,366	14,406
(1) Equipment	278	278	797	649	649	1173	927	927	1969
(2) Diesel	5744	5744	6364	3695	3695	6074	9439	9439	12,437
Irrigation	4169	35,920	5004	8248	19,820	7412	12,416	55,740	12,416
(1) Well–water	147	1287	177	294	710	264	441	1996	441
(2) Electricity	4022	34,633	4827	7954	19,111	7148	11,975	53,743	11,975
Fertilizer	15,310	15,310	15,310	15,310	15,310	15,310	30,619	30,619	30,619
(1) Nitrogen (N)	13,635	13,635	13,635	13,635	13,635	13,635	27,270	27,270	27,270
(2) Phosphate (P_2_O_5_)	1271	1271	1271	1271	1271	1271	2542	2542	2542
(3) Potash (K_2_O)	404	404	404	404	404	404	807	807	807
Plant protections	354	354	354	608	608	341	962	962	695
(1) Herbicide	242	242	242	496	496	229	738	738	471
(2) Insecticide	83	83	83	83	83	83	166	166	166
(3) Fungicide	29	29	29	29	29	29	58	58	58
Plastic film			3634			3002			6636
Labor	185	294	285	180	253	272	365	548	557
Total	28,395	60,255	34,102	29,029	40,675	33,922	57,424	100,930	68,024
**Output**									
Grain yield	61,489	134,681	101,090	123,517	150,444	146,168			

C, conventional rain–fed flat planting; WI, well-irrigation planting; RP, ridge-furrow planting with plastic film mulch over the ridge. Data are averaged over the two growing cycles.

The total energy inputs of maize production were 29,029, 40,675 and 33,922 MJ ha^−1^ under the control, WI, and RP, respectively (Table 2). The energy inputs of irrigation, fertilizers, and farm machinery were the main contributors, and occupied 28.4%, 52.7%, and 15.0% under the control, 48.7%, 37.6%, and 10.7% under WI, and 21.9%, 45.1%, and 21.4% under RP respectively. As to the entire rotation cycle, the total energy inputs were 57,424, 100,930, and 68,024 MJ ha^−1^ under the control, WI, and RP, respectively (Table 2).

**Figure 3:**
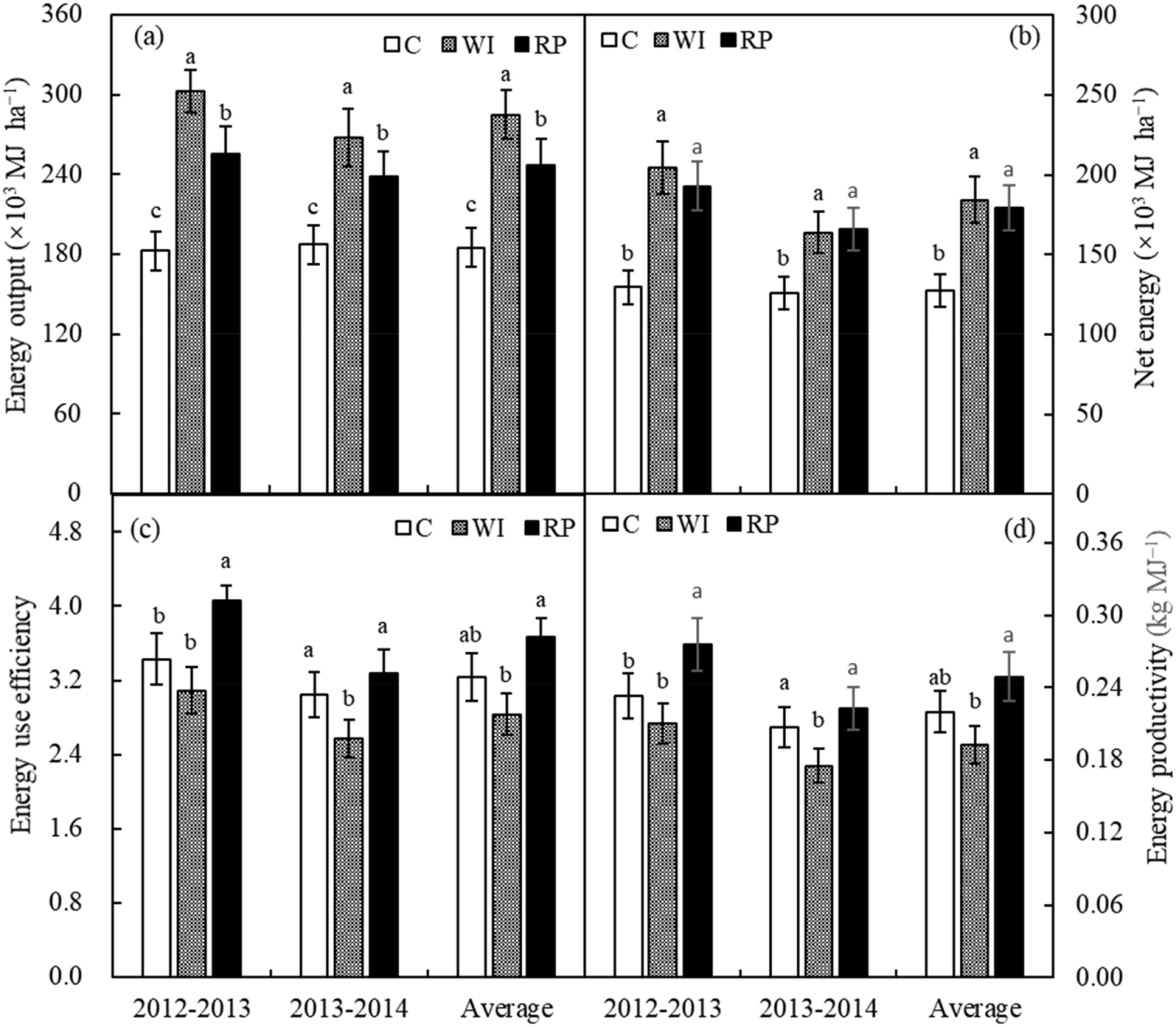
Effect of different planting strategies on energy output (**a**), net energy output (**b**), energy use efficiency (**c**), and energy productivity (**d**) of wheat–maize cropping system.

The annual average energy output from wheat and maize grains under RP was up to 101,090 MJ ha^–1^ and 146,168 MJ ha^–1^, respectively, which was visibly higher by 64.4% and 18.3% than that under the control, while lower by 24.9% and 2.8% than that under WI (Table 2), respectively. As to the entire rotation cycle, the annual average energy outputs of crop production under RP increased by 33.6% relative to that under the control, while reduced by 13.3% relative to that under the WI (Fig. 3a). The energy output under RP was significantly higher than those under the control, while lower than those under WI in 2012–2013 and 2013–2014, respectively (Fig. 3a). The net energy output under RP was sharply enhanced by 48.9% and 31.8% relative to those under the control in 2012–2013 and 2013–2014, respectively, while had no significant difference with those under WI over 2 rotation cycles (Fig. 3b). The energy use efficiency under RP was higher by18.3% and 7.5% than those under the control, and by 31.2% and 27.0% than those under WI in 2012–2013 and 2013–2014, respectively (Fig. 3c). Meanwhile, the energy productivity had the same trends with the energy use efficiency (Fig. 3d).

### Carbon footprint

The annual average CF under RP was obviously higher by 30.9% and 23.8% than those under the control for wheat and maize production, respectively (Table 3). However, there existed no significant difference between WI and RP for maize production, and a 15.4% reduce was found under WI for wheat production (Table 3). The annual average CF under RP increased by 27.2% relative to the control, while reduced by 6.8% relative to the WI in the entire rotation cycle (Table 3). The 165 and 1908 kg CO_2_–eq ha^–1^ was more from uses of farm machinery and plastic film under RP than those under both the control and WI, while 2785 kg CO_2_–eq ha^–1^ was less from uses of electricity for irrigation under RP than that under WI. Over 2 rotation cycles, the use of fertilizers and electricity for irrigation occupied 36.6% and 33.4% of the total emissions, followed by N_2_O emissions based on estimation (20.8%).

**Table 3:** Effect of different planting patterns on GHG emissions (kg CO_2_–eq ha^–1^) of wheat–maize cropping system.

Particulars	Wheat period			Maize period			The entire rotation cycle		
	C	WI	RP	C	WI	RP	C	WI	RP
Seeds	60	60	60	83	83	83	143	143	143
Farm machinery	316	316	350	203	203	334	520	520	685
Electricity	268	2309	322	530	1274	477	798	3583	798
Fertilizer	1964	1964	1964	1964	1964	1964	3928	3928	3928
(1) Nitrogen (N)	1868	1868	1868	1868	1868	1868	3735	3735	3735
(2) Phosphate (P_2_O_5_)	70	70	70	70	70	70	140	140	140
(3) Potash (K_2_O)	27	27	27	27	27	27	53	53	53
Plant protections	30	30	30	47	47	29	78	78	60
(1) Herbicide	16	16	16	34	34	16	50	50	32
(2) Insecticide	8	8	8	8	8	8	16	16	16
(3) Fungicide	6	6	6	6	6	6	11	11	11
Plastic film			1045			863			1908
Labor	81	129	125	79	111	119	160	240	244
Total N_2_O	1091	1091	1091	1139	1139	1139	2230	2230	2230
(1) Direct N_2_O^a^	745	745	745	745	745	745	1491	1491	1491
(2) Indirect N_2_O–1^b^	137	137	137	155	155	155	292	292	292
(3) Indirect N_2_O–2^c^	209	209	209	239	239	239	447	447	447
Carbon footprint	3811	5899	4988	4046	4822	5009	7857	10,721	9996

C, conventional rain–fed flat planting; WI, well-irrigation planting; RP, ridge-furrow planting with plastic film mulch over the ridge.

^a^Direct N_2_O, direct N_2_O emission from N fertilizer on upland crops.

^b^Indirect N_2_O–1, indirect N_2_O emission from synthetic N fertilizer volatilization.

^c^Indirect N_2_O–2, indirect N_2_O emission from N fertilizer leaching.

**Figure 4:**
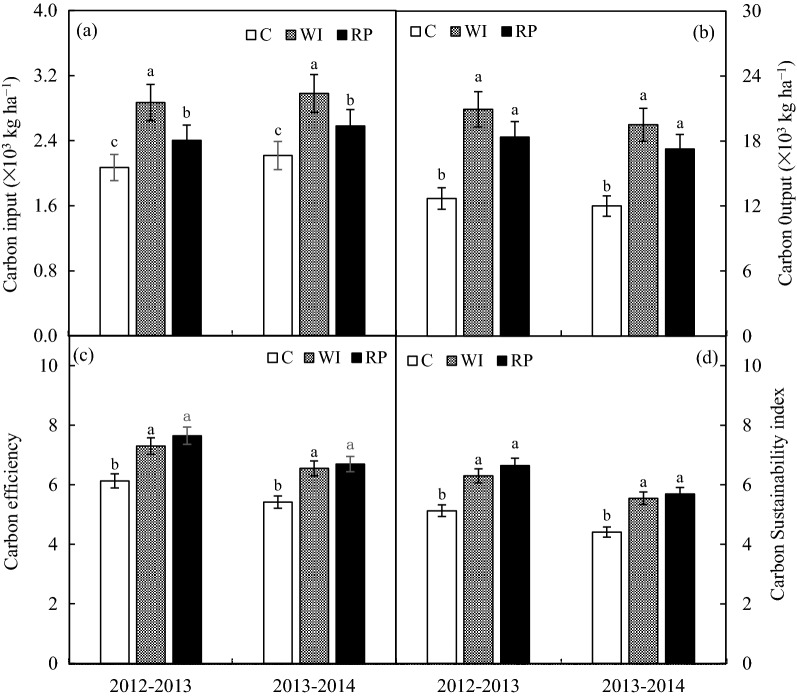
Effect of different planting strategies on carbon input (**a**), carbon output (**b**), carbon efficiency (**c**) and carbon sustainability index (**d**) of wheat–maize cropping system.

The carbon input under RP was significantly higher by 16.1% and 16.4% than those under the control, while lower by 16.2% and 13.5% than those under WI in 2012–2013 and 2013–2014, respectively (Fig. 4a). The carbon output under RP was significantly higher by 44.8% and 43.9% than those under the control, while lower by 12.3% and 11.5% than those under WI in 2012–2013 and 2013–2014, respectively (Fig. 4b). Meanwhile, the carbon efficiency under RP was significantly higher by 24.7% and 23.7% than those under the control, and slightly higher by 4.7% and 2.2% than those under WI in 2012–2013 and 2013–2014, respectively (Fig. 4c). Additionally, the carbon sustainability index under RP was significantly higher by 29.6% and 29.0% than those under the control, and slightly higher by 5.5% and 2.6% than those under WI in 2012–2013 and 2013–2014, respectively (Fig. 4d).

## Discussion

**Figure 5:**
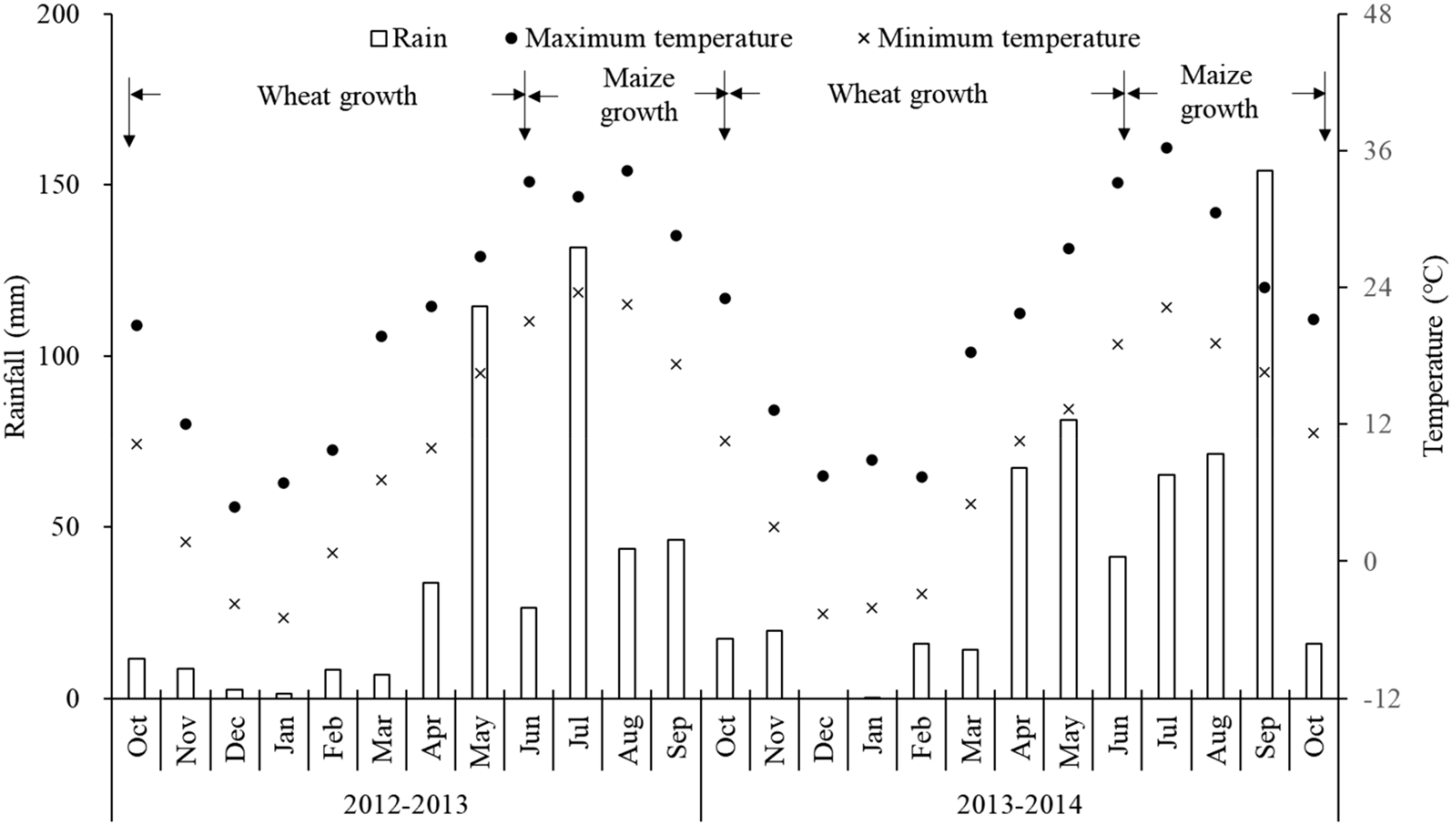
Monthly rainfall and mean temperature during crop growing season.

In the present study, significantly higher grain yields for both wheat and maize were achieved under RP than those under the control in both years (Fig. 1). However, remarkable reduction was only found for wheat grain yields when compared with the WI over the 2 rotation cycles (Fig. 1). Those results implied that adopting the RP could substantially promote grain yields under the wheat–maize cropping system in dry semi–humid areas, and that maize yields under RP reached a plateau close to the yield potential ceiling without drought stress^27^. The high grain yields under RP were mainly attributed to the superiority of RP in adjusting soil moisture and temperature to match the needs of crop production^17^. Similar results are also reported by Hu et al.^28^ in sub-humid drought-prone and semi–arid regions. Additionally, the maize yields in 2014 with a rainfall of 331 mm did not show any improvement over those in 2013 with a rainfall of 219 mm, although the rainfall increased by 51.1%. This phenomenon was mainly because the larger rainfall before the silking stage in 2013 (Fig. 5), resulting in a dramatically higher soil water storage to promote maize growth than those in 2014^23^. What’s more, more solar radiation for improving maize photosynthesis and growth, because the rainy days after silking in 2013 were lower than that in 2014.

The total cost of wheat production ranged from 6.266 × 10^3^ Yuan ha^–1^ under the control to 10.466 × 10^3^ Yuan ha^–1^ under WI (Table 1), falling well within the range of 2.402 –10.814 × 10^3^ Yuan ha^–1^ for wheat production reported by recent studies in China^10,29,30^. Likewise, the total cost of maize production ranged from 8.276 × 10^3^ Yuan ha^–1^ under the control to 10.076 × 10^3^ Yuan ha^–1^ under WI (Table 1), which also fell well within the range of 3.185–11.925 × 10^3^ Yuan ha^–1^ reported by Zheng et al.^31^ and Liang et al.^32^. Regarding the entire rotation cycle of wheat and maize, the total cost under RP was lower than that under WI. Those phenomena indicated that adopting the RP could reduce the cost of production when compared with the acknowledged high-yield production strategy of WI. Cost incurred for different component of cost analysis for the RP followed the order of farm machinery > fertilizer > labour > plastic film > seeds > irrigation/plant protections (Table 1). The order and share of different components were changed under the control and WI, because of the changes in costs involved in farm machinery, plastic film, irrigation, and labour. Due to the adoption of supplemental irrigation and water–saving measures, the gross returns under the WI and RP were significantly higher than those under the control throughout the two rotation cycles (Fig. 2). The gross return under the control was in proximity to the total economic production gained in the relative drought years, but lower than those in the relatively humid years reported by Lu and Liao^10^. However, the gross returns under WI and RP were also higher than those achieved by Lu and Liao^10^, whether in drought or a humid year. The gross returns from the RP were similar to that (38.122 × 10^3^ Yuan ha^–1^) reported under irrigated plots by Cui et al.^29^. Similarly, the net returns under the control in our study throughout the two rotation cycles were significantly lower than those from the WI and RP, and were below the net return values reported by Lu and Liao^10^. These results mainly attributed to the lower rainfall in our study. The net returns under the WI and RP in 2012–2013 also exceeded those gained under irrigated plots by Cui et al.^29^, but the net returns in 2013–2014 had a contrary tendency. The reason for those phenomena might be that the rainfall was not in step with crop growth needs in 2013–2014 (Fig. 5). The ratio of benefit: cost under RP was visibly higher than that under the control in 2012–2013, but did not differ with other treatments over 2 rotation cycles. Consequently, the results confirmed that adopting ridge-furrow planting with plastic film mulch over the ridge was a promising and economical option substitution for supplemental irrigation to produce wheat and maize grain in a dry semi–humid area of China.

The study has showed that the annual energy inputs of wheat production were ranged from 28,395 to 60,255 (Table 2). However, the total energy inputs of wheat production varied from 10,800 MJ ha^−1^ to 57,800 MJ ha^−1^ in other studies^8,33,34^. The values has exceeded the reported total energy inputs of wheat production due to the energy inputs from irrigation under WI (Table 2). In previous studies, the energy inputs of irrigation, nitrogen fertilizers, and farm machinery accounted for 23.5–32.1%, 24.0–38.3%, and 30.8–60.2% of the total energy inputs for raising wheat^34–36^. But the highest energy inputs under WI, control and RP were irrigation, fertilizer and fertilizer, respectively, which occupied over 40% of total energy inputs of wheat production. In addition, the use of plastic film contributed more than 10% to the total energy inputs under RP. The apparent discrepancy may result from different irrigation strategies and other field managements as well as edaphic and climatic conditions. The total energy inputs of maize production in the study were fairly high compared to other studies of 4200–10,400 MJ ha^−1^ in Bertocco et al.^37^ and of 12,700–23,000 MJ ha^−1^ in Amaducci et al.^38^. Similar to wheat production, irrigation, fertilizers, and farm machinery were also the main contributors of the energy inputs. In the entire rotation cycle, the total energy inputs showed: WI > RP > control (Table 2), which revealed that the total energy inputs of crop production under RP increased by 18.5% relative to that under the control, while reduced by 32.6% relative to that under the WI. Furthermore, the energy input derived from the irrigation is on the increase due to the decline of groundwater level^39^. This condition approved that adopting energy-save irrigation strategies, such as the ridge-furrow planting with plastic film mulch over the ridge, is urgent to supersede the supplemental irrigation to produce wheat and maize grain in a dry semi–humid area of China.

Values for energy output from wheat grains under RP and WI in the present study were higher than those previously reported values^34,40^, which was mainly due to the higher grain yields under RP and WI. Meanwhile, The obtained net energy outputs under RP and WI were higher than that reported by Singh et al.^41^. Additionally, the energy use efficiency and energy productivity under RP was higher than those under the control and WI in the entire rotation cycle. but the specific energy under RP was lower than those under the control and WI. Those results implied that adopting the RP could reduce direct energy input, offsetting the decreased system productivity and energy output from grain yield, and that adopting the RP can be expected to achieve identical results with those under well-irrigation planting in dry semi–humid regions due to better soil water conservation^23,42^.

As to the entire rotation cycle, the annual average CF showed: WI > RP > control (Table 3). The primary factors triggering significant differences in the CF among planting strategies were the different uses of farm machinery, plastic film, and electricity for irrigation. The use of fertilizers and electricity for irrigation occupied over 30% of the total emissions under two rotation cycles, which differed from the concept that 75.0% of GHG emissions derived from N fertilizer application during crop production^43,44^. This discrepancy could be because the Loess Plateau of China is susceptible to water scarcity with the evapotranspiration significantly exceeds the available precipitation^45^. Thus, electricity consumption for irrigation water from low groundwater levels per unit amount is larger than other regions. A similar result was also found in the North China Plain^4^. Thus, the RP can be considered as a viable planting strategy for practicing low-carbon agriculture in a dry semi–humid area of China.

The carbon input and carbon output under RP was significantly higher than those under the control, while lower than those under WI in two rotation cycles. Those results indicated the higher input produced more carbon output. For anthropogenic GHG emissions and their resulting global climate change, the sustainability of crop production increases with the increasing use efficiency of Carbon–based inputs^12^. The carbon efficiency and carbon sustainability index under RP was significantly higher than those under the control, and slightly higher than those under WI in two rotation cycles (Fig. 4); which exhibited that the RP was an effective substitution for supplemental irrigation for the mitigation of climate change and the achievement of sustained agricultural development in an intensive maize–wheat cropping system in a dry semi–humid area of China.

Although our study indicated that RF practice have lower carbon footprint and higher carbon efficiency, the use of plastic film can cause a series of environmental problems, for example white pollution, microplastic pollution and soil pollution^46^. After the plastic film was used in farmland, the plastic film cannot be completely removed and recycled and most of it remain in the soil for long time^47^. Which affects soil structure and mechanical tillage, resulted in environment pollution and mechanical damage. With the rapid promotion and application of plastic film in China, plastic film was covered in 19 million ha cropland and reached 2.7 million tons^48^. Fortunately, biodegradable film has similar properties to plastic film and reduce polyethylene residue in soil and plastic pollution^46^. This can be a good option to alternative plastic film and worth futher study for agricultural sustainable development and environmental protection. In addition, although the study and some others similar studies accomplished over a 2-years period^49–51^, some studies are more than 2 years, such as 4 or 6 years^1,20^, to reduce the effect of weather variability from year to year on crop growth, yield, irrigation and energy budget, carbon footprint^1,20^. Thus, this study needs to be conducted over a long period of time for further refine the results.

## Conclusions

This 2-year study assessed the impacts of different planting strategies on productivity, economic benefit, energy consumption and carbon footprint in an intensive wheat–maize cropping system to identify carbon friendly and cleaner planting technologies in a dry semi–humid area of China. The data showed that grain yields ranged from 3.22 to 9.31 Mg ha^−1^ for wheat and from 7.6 to 11.6 Mg ha^−1^ for maize, respectively, with the lowest yields under the control, followed by RP and WI. The gross return and net return had the same trends as those of grain yields, but the benefit: cost ratio was close between the WI and RP. The RP increased the net energy output, energy use efficiency, and energy productivity, but reduced the specific energy relative to the control. The annual average CF under RP increased by 27.2% relative to the control, while reduced by 6.8% relative to the WI. The carbon output under RP was significantly higher by 44.8% and 43.9% than those under the control, while slightly lower by 12.3% and 11.5% than those under WI in 2012–2013 and 2013–2014, respectively. The RP had the largest carbon efficiency and carbon sustainability. Therefore, shifting from planting strategies with supplemental irrigation to the ridge-furrow planting with plastic film mulch over the ridge increases the energy use efficiency and carbon efficiency, and thus provides potential solutions for the development of C–friendly planting technologies in dry semi-humid areas of China or other countries with similar agro–meteorology in the world. Nevertheless, the environment hazards of ridge-furrow planting with plastic film mulch over the ridge also needs to be concerned, for example, “white pollution” from plastic film. The innovation of covering material development and the formulation of related policies urgently need to solve this problem for better agricultural environment.

## Methods

### Experimental site and climate

The experiment was conducted at the Doukou Experimental Station of Northwest A&F University (34°36′N, 108°52′E) from October 2012–October 2014 in Sanyuan, Shaanxi Province, China. The study area has a temperate, dry semi–humid continental monsoon climate liable to drought with hot summers and cold winters. Based on 30 years’ climatic data, the annual average sunshine duration, temperature, and frost-free period was 2096 h, 13.4 °C, and 215 d, respectively. The annual average rainfall was 517.7 mm with 75% occurring from July to September. Precipitation data were recorded using standard weather station (Vantage Pro2, USA) on the experimental site. The daily maximum/minimum air temperature and precipitation distribution during experimental period are presented in Fig. 5. The amounts of precipitation were 183 and 222 mm during wheat growing season, and were 219 and 331 mm during maize growing season in 2012–2013 and 2013–2014 rotation cycles, respectively. The soil is classified as loamy clay^23^. The initial soil (0–20 cm) contained 17.77 g kg^−1^ SOM, 1.26 g kg^−1^ total N, 259.48 mg kg^−1^ available K, 22.08 mg kg^−1^ Olsen P with a pH of 8.45 (soil/water = 1:1) and a bulk density of 1.20 g cm^−3^.

### Experimental details

The field experiment included: conventional rain-fed flat planting (control, C), well-irrigation planting (WI), and ridge-furrow planting with plastic film mulch over the ridge (RP); the detail description was in Li et al.^23,52^. The treatments were applied in 6.4 m × 8 m plots in a randomized complete block design with four replications. The ridge-furrow planting systems were built by changing soil surface into alternating ridges and furrows with 30 and 55 cm in width. The ridges’ height was nearly 15 cm. The crops were sown in two rows in the furrows. The cultivars of wheat and maize were Xinong 979 and Zhengnong 9.

To ensure better seedling establishment, the control and RP plots were irrigated with 980 and 1180 m^3^ ha^−1^ at 8 days after sowing (DAG) during the second wheat period, and with 980 and 880 m^3^ ha^–1^ at 12 DAG during the first maize period and 3 days after sowing during the second maize period, respectively. No other supplemental irrigation was performed under control and RP plots. The WI plots were irrigated with 1200, 1100, 1100 and 1000 m^3^ ha^−1^ at 6, 89, 153 and 179 DAG during the first wheat period, with 1180, 1100, 1000 and 1000 m^3^ ha^−1^ at 8, 95, 160, and 180 DAG during the second wheat period, with 980 and 1000 m^3^ ha^−1^ at 12 and 50 DAG during the first maize period, and with 980, 790 and 980 m^3^ ha^−1^ at 3, 33 and 49 DAG during the second maize period, respectively.

During the wheat and maize periods, all of the treatments were fertilized with 90 kg N ha^−1^ and 50 kg P ha^−1^ and 30 kg K ha^−1^ by hand via broadcasting before sowing and then incorporated into the 0–20 cm soil layer with rotary tillage. Additionally, the plots were treated with 67.5 kg N ha^−1^ during the elongation and heading stages of wheat, and the elongation and tasseling stages of maize, respectively. The N topdressing was performed before raining or irrigation. All of wheat and maize straw were smashed (< 10 cm long) with a residue chopper after harvested with combine-harvesters. The chopped straw was incorporated into the soil by rotary tillage before ridge-furrow tillage. Other field management practices, including field preparation, sowing, harvesting, and the application of insecticides, herbicides and fungicides, followed the locally recommended practice in both years. The inputs are shown in Table S1.

### Yield measurements

At maturity, maize and wheat grains were manually harvested in duplicate from the center (6 and 2 m^2^ for each crop) of each plot every year. After air-drying, portions of grain were oven-dried at 60 °C for grain determination. System productivity in term of wheat equivalent yields (WEY) was estimated to compare the effects of different treatments on crop performances by converting grain yields of both crops into the WEY on the basis of market price followed with the Eq. (1):

1 $$\mathrm{WEY}=\mathrm{Wheat} \; \text{yield}+\left(\mathrm{Maize} \; \text{yield}\times \frac{{\mathrm{M}}_{\mathrm{p}}}{{\mathrm{W}}_{\mathrm{p}}}\right)$$

where WEY is the system productivity; M_p_ and W_p_ are the market price of maize and wheat grains. During the study period, the annual average maize and wheat grain prices were 2.40 and 2.06 Yuan kg^–1^, respectively.

### Economic analysis

The economic analysis was computed by assessing a range of components, including the cost of cultivation (C_tot_), gross revenue (GR), economic profit (EP), and the ratio of net income to cost (RIC). These analyses were conducted based on the prevailing market price of the inputs, outputs, and services, and were followed with the equations [Eqs. (2)–(5)] suggested by Lu and Liao^10^.

2 $${\mathrm{C}}_{\mathrm{tot}}=\sum_{\mathrm{i}=1}^{\mathrm{n}}\frac{\mathrm{C}1+\mathrm{C}2+\cdots \mathrm{Ci})}{1000}$$

where, C_tot_ is the total cost (× 10^3^ Yuan ha^−1^) for each treatment. C_1_, C_2_… C_i_ is the cost (Yuan ha^−1^) of input i (i = 1–13, Table S1).

3 $$\mathrm{GR}=\frac{\mathrm{Y}\times \mathrm{P}}{1000}$$

where, GR is the gross revenue (× 10^3^ Yuan ha^−1^). Y is the grain yields (Mg ha^−1^, OW). P is the corresponding prevailing market grain prices (Yuan kg^−1^).

4 $$\mathrm{EP}=\mathrm{GR}-{\mathrm{C}}_{\mathrm{tot}}$$

where, EP is economic profit (net income, × 10^3^ Yuan ha^−1^).

5 $$\mathrm{RIC}=\frac{\mathrm{EP}}{\mathrm{Cost}}$$

where, RIC is the ratio of net income to cost.

### Energy analysis

The energy inputs and outputs of each treatment were estimated based the complete record of all inputs (Table S1) and outputs (grain yields).

The inputs and outputs were computed from physical units to energy units through multiplication with the conversion coefficients (Table S2). The energy input (EI) and output (EO), net energy output (NEO), energy use efficiency (EUE), energy productivity (EP) were calculated by Eqs. (6)–(10)^1^.

6 $$\mathrm{EI}=\sum_{\mathrm{i}=1}^{\mathrm{n}}\left(\mathrm{C}1+\mathrm{C}2+\cdots \mathrm{Ci}\right)$$

where, EI is the total energy input (MJ ha^−1^). C_1_, C_2_… C_i_ is the energy input (MJ ha^−1^) of i (i = 1–13, Table S1).

7 $$\mathrm{EO}=\mathrm{Y}\times \mathrm{EC}$$

where, EO is the total energy out (MJ ha^−1^). Y is the grain yields (Mg ha^−1^, OW). EC is the corresponding energy coefficient of grain yields.

8 $$\mathrm{NEO}=\mathrm{EO}-\mathrm{EI}$$

where, NEO is net energy out (MJ ha^−1^).

9 $$\mathrm{EUE}=\frac{\mathrm{EO}}{\mathrm{EI}}\times 100\mathrm{\%}$$

where, EUE is the energy use efficiency (%).

10 $$\mathrm{EP}=\frac{\mathrm{WEY}}{\mathrm{EI}}$$

where, EP is the energy productivity. WEP is the system productivity.

### Carbon footprint (CF)

The CF was been used to assessed environmental impacts of different planting patterns, because the CF can be as a powerful tool to know and build more environmentally friendly crop production systems^53,54^. The CF is the total amount of GHG emissions (CO_2_ and N_2_O, CO_2_ equivalents) throughout the crop growth^55^. Because of CH_4_ emission was often negligible in dry semi-humid regions, our recent study only considered the N_2_O and CO_2_ gases. The N_2_O was converted into 265 CO_2_ equivalents^3^. The corresponding emission coefficients, which was presented in Table S3, were used to calculated the GHG emissions of the field operation and inputs. In fields, ammonia volatilization was determined from fertilizer-N using rates of 23% and 26% for wheat and maize, respectively^56^. Nitrate leaching was determined from fertilizer-N using rates of 14% and 16% for wheat and maize, respectively^43^. Direct N_2_O emissions came from 1.25% of fertilizer-N^56^. Indirect N_2_O emissions were estimated by 1% of ammonia–N and 2.5% of nitrate–N, respectively^56^. The carbon footprints (CF, kg CO_2_–eq ha^–1^) was obtained using Eq. (11):

11 $$\mathrm{CF}={\mathrm{N}}_{2}\text{O} \; \text{emission}\times 265+{\mathrm{CO}}_{2} \;\mathrm{ emission}$$

where, CF is the energy productivity.

### Carbon output, carbon efficiency, and carbon sustainability index

The carbon output is the total carbon equivalent of grain, straw, stubble and root biomass produced by the crop^57^. The below–ground root biomass represented 22% and 23% of wheat and maize straw biomass, respectively^58^. The proportions of stubble to straw biomass were estimated to be 20% and 10% for wheat and maize, respectively. The carbon content was assumed to be 40% for both wheat and maize biomasses. Carbon efficiency was calculated as the ratio of carbon output to carbon input, and the carbon sustainability index was estimated by computing the difference between carbon output and carbon input and dividing it by carbon input^1,12,59,60^.

### Statistical analysis

Statistical analyses were performed by using Excel 2013 and SPSS 19.0 (SPSS Inc., Chicago, IL, US). The mean differences among treatments were determined by the Duncan multiple range test at P < 0.05.

### Statement

The authors declare that our field studies comply with China’s guidelines and legislation.

## Supplementary Information

Supplementary Information.
