# Supplementary material for: Energy budget and carbon footprint in a wheat and maize system under ridge furrow strategy in dry semi humid areas
Source: Sci Rep. 2021 Apr 30;11:9367. doi: 10.1038/s41598-021-88717-3 (PMC8087763; doi:10.1038/s41598-021-88717-3)
Supplement: Supplementary file 1 — Supplementary Information. [file 41598_2021_88717_MOESM1_ESM.docx]

**Energy budget and carbon footprint in a wheat and maize system under ridge furrow strategy in dry semi humid areas**

Changjiang Li ^1, 3^, Shuo Li ^2, *^

^1^ Hainan Key Laboratory for Sustainable Utilization of Tropical Bioresources, College of Tropical Crops, Hainan University, Haikou 570228, China; [lichangjiang99@163.com](mailto:lichangjiang99@163.com) (C.L.)

^2^ College of Life Sciences, Hebei University, Baoding 071000, China

^3^ College of Agronomy, Northwest A&F University, Yangling 712100, China

*Correspondence: [shuol9011@126.com](mailto:shuol9011@126.com;) (S.L.)

| Particulars | Wheat period | | |  | Maize period | | |
| --- | --- | --- | --- | --- | --- | --- | --- |
|  | C^a^ | WI | RP |  | C | WI | RP |
| Seeds (kg ha^–1^ yr^-1^) | 150.00 | 150.00 | 150.00 |  | 21.60 | 21.60 | 21.60 |
| Machinery (kg ha^–1^ yr^-1^)^b^ | 4.44 | 4.44 | 12.70 |  | 10.35 | 10.35 | 18.70 |
| Diesel (kg ha^–1^ yr^-1^) | 102.01 | 102.01 | 113.01 |  | 65.62 | 65.62 | 107.86 |
| Well–water (m^3^ ha^–1^ yr^-1^) | 490.00 | 4290.00 | 590.00 |  | 980.00 | 2365.00 | 880.00 |
| Electricity (kWh ha^–1^ yr^-1^) | 335.13 | 2886.05 | 402.26 |  | 662.81 | 1592.55 | 595.68 |
| Human power (h ha^–1^ yr^-1^) | 94.40 | 150.24 | 145.47 |  | 92.00 | 129.15 | 138.53 |
| Nitrogen (N, kg ha^–1^ yr^-1^) | 225.00 | 225.00 | 225.00 |  | 225.00 | 225.00 | 225.00 |
| Phosphate (P_2_O_5_, kg ha^–1^ yr^-1^) | 114.52 | 114.52 | 114.52 |  | 114.52 | 114.52 | 114.52 |
| Potash (K_2_O, kg ha^–1^ yr^-1^) | 60.26 | 60.26 | 60.26 |  | 60.26 | 60.26 | 60.26 |
| Herbicide (kg ha^–1^ yr^-1^) | 0.95 | 0.95 | 0.95 |  | 1.95 | 1.95 | 0.90 |
| Insecticide (kg ha^–1^ yr^-1^) | 0.45 | 0.45 | 0.45 |  | 0.45 | 0.45 | 0.45 |
| Fungicide (kg ha^–1^ yr^-1^) | 0.30 | 0.30 | 0.30 |  | 0.30 | 0.30 | 0.30 |
| Plastic film (kg ha^–1^ yr^-1^) |  |  | 46.00 |  |  |  | 38.00 |

**Table S1.** Agriculture inputs used under different planting strategies in wheat–maize cropping system. ^a^C, control, that is, conventional rain–fed flat planting; WI, well-irrigation planting; RP, ridge-furrow planting with plastic film mulch over the ridge. ^b^Machinery included tractors, rotary cultivator, ridger, seeder, fertilizer applicator, harvester, and the agriculture inputs of machineries were converted according to the service life of 20 years. The same in subsequent figures and tables.

| Particulars | Units | Energy equivalent (MJ unit^–1^) | References |
| --- | --- | --- | --- |
| **Input** |  |  |  |
| Human labor | Man–hour | 1.96 | Singh et al.^42^ |
| Machinery | kg | 62.70 | Singh et al.^42^ |
| Diesel | kg | 56.31 | Singh et al.^42^ |
| Nitrogen (N) | kg | 60.60 | Singh et al.^42^ |
| Phosphorus (P_2_O_5_) | kg | 11.10 | Singh et al.^42^ |
| Potassium (K_2_O) | kg | 6.70 | Singh et al.^42^ |
| Herbicide | kg | 254.45 | Singh et al.^42^ |
| Insecticide | kg | 184.63 | Singh et al.^42^ |
| Fungicide | kg | 97.00 | Singh et al.^42^ |
| Plastic film | kg | 79.00 | Yuan et al.^42^ |
| Well–water | m^3^ | 0.30 | Nasseri^35^ |
| Electricity | kWh | 12.00 | Nasseri^35^ |
| Wheat/maize seeds | kg | 15.70 | Singh et al.^42^ |
| **Output** |  |  |  |
| Wheat/maize grain | kg | 14.70 | Singh et al.^42^ |
| Wheat/maize straw | kg | 12.50 | Singh et al.^42^ |

**Table S2.** Energy equivalents used for energy input and output calculations.

| Particulars | Emission factor | References |
| --- | --- | --- |
| Human labor | 0.86 kg CO_2_–eq d^–1^ | Zhu et al.^59^ |
| Diesel | 3.10 kg CO_2_–eq kg^–1^ | Zhu et al.^59^ |
| Nitrogen (N) | 8.30 kg CO_2_–eq kg^–1^ | Zhu et al.^59^ |
| Phosphorus (P_2_O_5_) | 0.61 kg CO_2_–eq kg^–1^ | Zhu et al.^59^ |
| Potassium (K_2_O) | 0.44 kg CO_2_–eq kg^–1^ | Zhu et al.^59^ |
| Herbicide | 17.24 kg CO_2_–eq kg^–1^ | Zhu et al.^59^ |
| Insecticide | 18.08 kg CO_2_–eq kg^–1^ | Zhu et al.^59^ |
| Fungicide | 18.98 kg CO_2_–eq kg^–1^ | Zhu et al.^59^ |
| Plastic film | 22.72 kg CO_2_–eq kg^–1^ | Wang et al.^60^ |
| Electricity | 0.80 kg CO_2_–eq kWh^–1^ | Zhu et al.^59^ |
| Maize seeds | 3.85 kg CO_2_–eq kg^–1^ | Zhu et al.^59^ |
| Wheat seeds | 0.40 kg CO_2_–eq kg^–1^ | Zhu et al.^59^ |

**Table S3.** Emission factors of agriculture inputs used in the estimation.
